# Mass Cytometry Identifies Distinct Lung CD4^+^ T Cell Patterns in Löfgren’s Syndrome and Non-Löfgren’s Syndrome Sarcoidosis

**DOI:** 10.3389/fimmu.2017.01130

**Published:** 2017-09-12

**Authors:** Ylva Kaiser, Tadepally Lakshmikanth, Yang Chen, Jaromir Mikes, Anders Eklund, Petter Brodin, Adnane Achour, Johan Grunewald

**Affiliations:** ^1^Respiratory Medicine Unit, Department of Medicine, Solna and Center for Molecular Medicine, Karolinska Institutet and Karolinska University Hospital, Stockholm, Sweden; ^2^Science for Life Laboratory, Department of Medicine, Karolinska Institutet, Stockholm, Sweden; ^3^Department of Infectious Diseases, Karolinska University Hospital, Stockholm, Sweden; ^4^Department of Neonatology, Karolinska University Hospital, Stockholm, Sweden

**Keywords:** sarcoidosis, mass cytometry, bronchoalveolar lavage, CD4^+^ T cells, Löfgren’s syndrome, granuloma, disease phenotypes

## Abstract

Sarcoidosis is a granulomatous disorder of unknown etiology, characterized by accumulation of activated CD4^+^ T cells in the lungs. Disease phenotypes Löfgren’s syndrome (LS) and “non-LS” differ in terms of clinical manifestations, genetic background, HLA association, and prognosis, but the underlying inflammatory mechanisms largely remain unknown. Bronchoalveolar lavage fluid cells from four HLA-DRB1*03^+^ LS and four HLA-DRB1*03^−^ non-LS patients were analyzed by mass cytometry, using a panel of 33 unique markers. Differentially regulated CD4^+^ T cell populations were identified using the Citrus algorithm, and *t*-stochastic neighborhood embedding was applied for dimensionality reduction and single-cell data visualization. We identified 19 individual CD4^+^ T cell clusters differing significantly in abundance between LS and non-LS patients. Seven clusters more frequent in LS patients were characterized by significantly higher expression of regulatory receptors CTLA-4, PD-1, and ICOS, along with low expression of adhesion marker CD44. In contrast, 12 clusters primarily found in non-LS displayed elevated expression of activation and effector markers HLA-DR, CD127, CD39, as well as CD44. Hierarchical clustering further indicated functional heterogeneity and diverse origins of T cell receptor Vα2.3/Vβ22-restricted cells in LS. Finally, a near-complete overlap of CD8 and Ki-67 expression suggested larger influence of CD8^+^ T cell activity on sarcoid inflammation than previously appreciated. In this study, we provide detailed characterization of pulmonary T cells and immunological parameters that define separate disease pathways in LS and non-LS. With direct association to clinical parameters, such as granuloma persistence, resolution, or chronic inflammation, these results provide a valuable foundation for further exploration and potential clinical application.

## Introduction

Sarcoidosis is a multisystem granulomatous disorder of unknown etiology, characterized by accumulation of activated CD4^+^ T cells in the lungs (1). Löfgren’s syndrome (LS) is distinguished by an acute disease onset with fever, bilateral hilar lymphadenopathy (BHL), erythema nodosum, and/or ankle arthritis, as well as a good prognosis and typically self-limiting disease course (2). In contrast, “non-LS” sarcoidosis constitutes a more heterogeneous patient group that is usually characterized by an insidious onset and disease progression, with substantial risk of developing chronic disease and pulmonary fibrosis. LS is associated with carriage of the HLA-DRB1*03 allele (3), which also strongly influences spontaneous disease resolution (4). The presence of clonal T cell receptor (TCR)-restricted cells in HLA-DRB1*03^+^ patients further implicates specific antigen recognition in the lungs (5, 6).

Detailed genetic profiling indicates that LS and non-LS share much fewer features than previously acknowledged (7), and arguments have been made for LS to be considered a separate disease entity. However, understanding of pulmonary immune pathways underlying the observed clinical heterogeneity remains incomplete and, until recently, methodology for adequately addressing this question has been lacking.

In this study, we, therefore, subjected bronchoalveolar lavage fluid (BALF) cells from four HLA-DRB1*03^+^ LS and four HLA-DRB1*03^−^ non-LS patients (Table 1), which are believed to represent two clinical opposites, to mass cytometry, utilizing a T cell panel of 33 unique markers. Unbiased data analysis was performed using two different clustering algorithms to identify differentially regulated cell populations. Specific attention was paid to the recently identified subpopulation preferentially expressing TCR variable segments Vα2.3 and Vβ22 in HLA-DRB1*03^+^ LS patients (8). With the aim of identifying novel cell populations and pathways that associate with disease resolution or progression, this study demonstrates the sensitivity of mass cytometry, its ability to detect significant differences between patient groups despite limited sample sizes, and the benefit of its application in complex diseases. Albeit in a small study population, we here present novel data on immunological markers that differentiate between sarcoidosis subtypes at the molecular level, and that can be targeted by future exploration in larger patient cohorts.

**Table 1:** Clinical characteristics of sarcoidosis patients.

	Among all sarcoidosis patients (*n* = 8)	Among Löfgren’s syndrome (LS) patients (*n* = 4)	Among non-LS patients (*n* = 4)
Sex (male/female)	7/1	3/1	4/0
Age, years	43.0 (33.0–50.0)	41.0 (33.0–50.0)	43.0 (40.0–46.0)
Chest radiographic stage 0/I/II/III/IV^a^	0/2/6/0/0	0/2/2/0/0	0/0/4/0/0
Smoking status (non-smoker/former/current)	4/3/1	1/2/1	3/1/0
VC (% of predicted)	84.5 (78.5–95.0)	89.0 (77.8–99.0)	84.5 (80.0–88.8)
DLCO (% of predicted)	95.0 (77.0–101.0)	103.0 (99.0–107.0)	77.0 (75.5–89.0)
FEV1 (% of predicted)	81.5 (73.8–86.8)	82.5 (78.3–86.8)	78.5 (73.5–85.5)
BALF cell concentration (10^6^ cells/l)	272.7 (164.0–325.2)	221.6 (163.9–316.6)	278.4 (240.7–339.5)
% BALF recovery	64.0 (62.8–69.3)	62.5 (61.3–64.8)	66.5 (64.0–70.5)
% BALF macrophages	77.9 (65.9–80.9)	82.4 (77.5–85.5)	62.6 (43.2–77.8)
% BALF lymphocytes	19.7 (13.5–32.9)	13.4 (12.3–17.0)	36.2 (20.7–54.3)
% BALF neutrophils	1.2 (0.9–4.3)	1.2 (0.9–3.8)	2.6 (0.8–4.3)
% BALF eosinophils	0.0 (0.0–0.1)	0.0 (0.0–0.1)	0.0 (0.0–0.1)
Bronchoalveolar lavage CD4/CD8 ratio	4.9 (4.0–7.7)	7.8 (2.7–14.9)	4.9 (4.7–5.2)
HLA-DRB1*03^+^/DRB1*03^−^DRB3*01^+^/DRB1*03^−^DRB3*01^−^	4/0/4	4/0/0	0/0/4
% Vα2.3^+^CD4^+^ T cells in BALF	12.0 (4.1–24.0)	25.3 (21.8–30.2)	3.8 (3.3–4.4)
% Vβ22^+^CD4^+^ T cells in BALF	4.3 (4.0–9.3)	9.3 (6.8–12.6)	3.2 (2.7–3.6)
% Vα2.3^+^Vβ22^+^CD4^+^ T cells in BALF	1.9 (0.5–6.0)	6.9 (5.0–9.2)	0.5 (0.4–0.6)

*^a^Chest radiography staging as follows: stage 0, normal chest radiography; stage I, enlarged lymph nodes; stage II, enlarged lymph nodes with parenchymal infiltrates; stage III, parenchymal infiltrates without enlarged lymph nodes; and stage IV, signs of pulmonary fibrosis*.

*VC, vital capacity; DLCO, carbon monoxide diffusing capacity; FEV1, forced expiratory volume in 1 s; BALF, bronchoalveolar lavage fluid*.

*All percentage values are denoted as median (p25–p75)*.

## Materials and Methods

### Study Subjects, Bronchoscopy, and Bronchoalveolar Lavage (BAL)

Bronchoscopy with BAL was performed as previously described (9). Eight newly diagnosed sarcoidosis patients (one female) with a median age of 43 years (Table 1) were included in the study. All patients were HLA-typed and diagnosed with sarcoidosis according to criteria established by the World Association of Sarcoidosis and Other Granulomatous Disorders (WASOG) (10). Specifically, these included typical clinical and radiographic manifestations, findings at bronchoscopy with BAL including an elevated CD4/CD8 ratio and, if required, positive biopsies, as well as exclusion of other diagnoses. Four patients were diagnosed with LS, defined as an acute onset, chest radiographic findings of BHL alone (*n* = 2), or BHL with pulmonary infiltrates (*n* = 2), and erythema nodosum and/or bilateral ankle arthritis. Four patients presented with non-LS sarcoidosis, with insidious onset, fatigue, dry cough, and parenchymal changes. In addition, BALF cells from one healthy volunteer and peripheral blood mononuclear cells (PBMCs) from three healthy donors were used as staining controls. Informed consent was obtained from all subjects and ethical approval granted from the Stockholm County Regional Ethical Committee (approval numbers: 2005/1031-31/2, 2009/20-32, and 2011/35-32).

### HLA Typing

Genomic DNA was extracted from whole blood samples of patients and healthy volunteers. HLA-DRB1 and DRB3 alleles were subsequently determined by the PCR-sequence-specific primer technique (Olerup SSP-DR Low Resolution Kit, Saltsjöbaden, Sweden) as previously described (11).

### Assessment of TCR Expression by Flow Cytometry

Freshly isolated BALF cells were stained *ex vivo* using the following antibodies: CD3-Pacific Blue, clone UCHT1 (BD Pharmingen, San Diego, CA, USA), CD4-APC-H7, clone SK3 (BD Biosciences, San Jose, CA, USA), Vα2.3-FITC, clone F1 (Thermo Scientific, Rockford, IL, USA), and Vβ22-PE, clone IMMU 546 (Beckman Coulter Immunotech, Marseille, France). Live/Dead Fixable Aqua Dead Cell Stain Kit (Life Technologies, Eugene, OR, USA) was used for assessment of cell viability. Cells were sequentially gated on lymphocytes (based on FSC vs. SSC), single cells (based on FSC-A vs. FSC-H), viable cells (defined as Aqua negatively stained cells), CD3^+^, and CD4^+^ cells. The CD4^+^ gate was set as threshold gate for acquisition, with a minimum of 15,000 events being collected. Flow cytometry was run on a BD FACSVerse (Beckton Dickinson, San Jose, CA, USA) and results were analyzed using FlowJo X (TreeStar, Ashland, OR, USA) software.

### Mass Cytometry

Cryopreserved BALF cells obtained from eight sarcoidosis patients (LS, *n* = 4; non-LS, *n* = 4) were thawed in RPMI medium supplemented with fetal bovine serum (FBS), penicillin–streptomycin, and benzonase (Sigma-Aldrich, St. Louis, MO, USA). BALF cells from one healthy individual and PBMCs from three healthy donors were included as staining controls but not subjected to bioinformatic analysis. Briefly, for live–dead cell distinction, cells were stained with 2.5 µM Cisplatin (Fluidigm, South San Francisco, CA, USA) in RPMI without serum for 5 min at RT and quenched with RPMI containing FBS. An Agilent robotic platform was used for staining cells, as this offers more uniform staining and less variability among samples. Cells were then re-suspended in CyFACS buffer (PBS with 0.1% BSA, 0.05% sodium azide, and 2 mM EDTA) and counted; approximately 1–2 million live cells were used for staining in a 96-well round-bottom plate. Next, cells were incubated for 30 min at 4°C with a 30 µl cocktail of metal-conjugated antibodies targeting surface antigens in a T cell panel (Table 2). Following wash with CyFACS buffer and overnight fixation using 1% formaldehyde made in PBS (Polysciences Inc., Warminster, PA, USA), cells were permeabilized using intracellular fixation and permeabilization buffer set (eBiosciences Inc., San Diego, CA, USA) according to the manufacturer’s recommendations and stained with 30 µl of intracellular antibody cocktail (Ki-67) for 60 min at RT. Cells were washed and fixed in 1% formaldehyde at 4°C until acquisition. On the day of acquisition (within a week after staining), cells were stained with DNA intercalator (0.125 µM Iridium-191/193 or MaxPar^®^ Intercalator-Ir; Fluidigm) in 1% formaldehyde made in PBS for 20 min at RT. After multiple washes with CyFACS, PBS, and MilliQ water, cells were filtered through a 35-µm nylon mesh and diluted to 500,000 cells/ml. Cells were acquired at a rate of 300–500 cells/s using a CyTOF2 mass cytometer (Fluidigm), CyTOF software v.6.0.626 with noise reduction, a lower convolution threshold of 200, event length limits of 10–150 pushes, a sigma value of 3, and a flow rate of 0.045 ml/min.

**Table 2:** Mass cytometry staining panel.

Marker	Tag	Clone	Vendor
CD45	89Y	HI30	Fluidigm
CD57	115In	HCD57	BioLegend
CD196 (CCR6)	141Pr	11A9	BD Pharmingen
CD19	142Nd	HIB19	Fluidigm
CD5	143Nd	UCHT2	Biolegend
CD195 (CCR5)	144Nd	NP-6G4	Fluidigm
CD4	145Nd	RPA-T4	Fluidigm
CD8a	146Nd	SK1	BioLegend
CD11c	147Sm	Bu15	Fluidigm
CD31	148Nd	WM59	BioLegend
CD278 (ICOS)	151Eu	DX29	Fluidigm
αβTCR	152Sm	IP26	BioLegend
CD3ε	154Sm	UCHT1	Fluidigm
CD194 (CCR4)	155Gd	205410	R&D Systems
Vα2.3	156Gd	F1	Thermo Scientific
CXCR3	157Gd	G025H7	BioLegend
Vβ22	159Tb	IMMU 546	Beckman Coulter Immunotech
CD28	160Gd	CD28.2	BioLegend
CD161	161Dy	HP-3G10	BioLegend
Ki-67	162Dy	B56	Fluidigm
HLA-DR	163Dy	L243	BioLegend
CD44	164Dy	BJ18	BioLegend
CD127	165Ho	A019D5	Fluidigm
CD27	167Er	L128	Fluidigm
CD38	168Er	HIT2	BioLegend
CD45RA	169Tm	HI100	Fluidigm
CD152 (CTLA-4)	170Er	14D3	Fluidigm
CD279 (PD-1)	172Yb	EH12.2H7	BioLegend
CD39	173Yb	A1	BioLegend
CXCR5	174Yb	51505	R&D Systems
Cell-ID™ Intercalator-Ir (DNA)	191Ir	–	Fluidigm
Cell-ID™ Intercalator-Ir (DNA)	193Ir	–	Fluidigm
Cell-ID™ Cisplatin (Live–dead)	195Pt	–	Fluidigm

*CD, cluster of differentiation; CTLA-4, cytotoxic T lymphocyte-associated protein 4; CCR, C–C chemokine receptor; ICOS, inducible co-stimulatory molecule; PD-1, programmed cell death protein 1; CXCR, CXC chemokine receptor; TCR, T cell receptor; HLA-DR, human leukocyte antigen-antigen D related; Sm, samarium; Nd, neodymium; Er, erbium; Gd, gadolinium; Yb, ytterbium; Dy, dysprosium; Y, yttrium; Tm, thulium; In, indium; Ho, holmium; Gd, gadolinium; Pr, praseodymium; Eu, europium; Pt, platinum; Ir, iridium*.

*Fluidigm, South San Francisco, CA, USA; BioLegend, San Diego, CA, USA; BD Pharmingen, San Diego, CA, USA; R&D Systems Inc., Minneapolis, MN, USA; Thermo Scientific, Rockford, IL. USA; Beckman Coulter Immunotech, Marseille, France*.

### Antibodies and Reagents

Purified antibodies were obtained in protein-free buffer and subsequently coupled to isotopically purified metals using MaxPar^®^ antibody conjugation kits (Fluidigm) (12), as per the manufacturer’s protocol. After determining protein concentration by measurement of absorbance at 280 nm, metal-labeled antibodies were diluted in Candor PBS Antibody Stabilization solution (Candor Bioscience, Wangen, Germany) for long-term storage at 4°C. Antibodies used in this study are listed in Table 2.

### Dimensionality Reduction, Cluster Analysis, and Visualization

Sarcoid BALF samples were sequentially gated on single cells, viable (DNA^+^Cisplatin^−^) cells, and CD3^+^CD4^+^CD8^−^ T cells. Based on the collective expression of the remaining 28 T cell-specific markers in the panel (Table 2), the Citrus algorithm (13) was used to identify differently abundant T cell populations in LS and non-LS samples, and clusters were visualized by ACCENSE (14). Citrus was run using the original R implementation (v. citrus_0.08) in R v.3.3.1 (2016-06-21) with a random down-sampling to 1,500 manually gated CD4^+^ T-cells from each file. The following markers were used for cluster analysis: CD45, CD57, CCR6, CD5, CCR5, CD4, CD8a, CD31, ICOS, TCRαβ, CD3e, CCR4, Vα2.3, CXCR3, Vβ22, CD28, CD161, Ki-67, HLA-DR, CD44, CD127, CD27, CD38, CD45RA, CTLA-4, PD-1, CD39, and CXCR5. The collective expression across all markers was used to define cell populations. The “pamr” model was applied for calculating differential regulation, using a relaxed FDR-constrained model for exploratory purposes (FDR < 0.25). Cells from all samples were combined and subjected to hierarchical clustering. Descriptive features of identified clusters were calculated on a per-sample basis to train a regularized regression model predictive of the two patient groups. Classification models were constructed using the nearest shrunken centroid and lasso-regularized logistic regression methods, both of which build a series of predictive models using automatically selected informative subsets of supplied regressors. Internal cross-validation was used to evaluate model fit and select an appropriate threshold for the final model.

Dimensionality reduction of unclustered data was performed using the *t*-stochastic neighborhood embedding (*t*-SNE) algorithm implemented in the Cytofkit library (15–17), supplied by Bioconductor v.3.4 (18, 19) and run in RStudio v.1.0.44 (2016-11-01) (20). The markers used for clustering were CD45, CD57, CCR6, CD5, CCR5, CD4, CD8a, CD31, ICOS, TCRαβ, CD3e, CCR4, Vα2.3, CXCR3, Vβ22, CD28, CD161, Ki-67, HLA-DR, CD44, CD127, CD27, CD38, CD45RA, CTLA-4, PD-1, CD39, CXCR5, CD19, and CD11c. A fixed number of 5,000 cells were sampled without replacement from each file and combined for analysis. Resulting *t*-SNE plots were subsequently filtered by marker expression and patient group to visualize differences between LS and non-LS.

Complementary analysis and validation of marker expression in BALF CD4^+^ T cells from LS and non-LS patients, respectively, as well as from a single healthy individual, were performed using FlowJo X (TreeStar) software, based on the above mentioned gating strategy [i.e., single cells, viable (DNA^+^Cisplatin^−^) cells, and CD3^+^CD4^+^CD8^−^ T cells]. Univariate statistical analysis of marker expression in LS vs. non-LS CD4^+^ T cells was performed using the non-parametric Mann–Whitney *U* test in GraphPad Prism v.5.02 software (GraphPad Software, Inc., La Jolla, CA, USA.). *p* < 0.05 was considered significant.

## Results

### Abundance of Individual CD4^+^ T Cell Clusters Differs Significantly between LS and Non-LS

**Figure 1:**
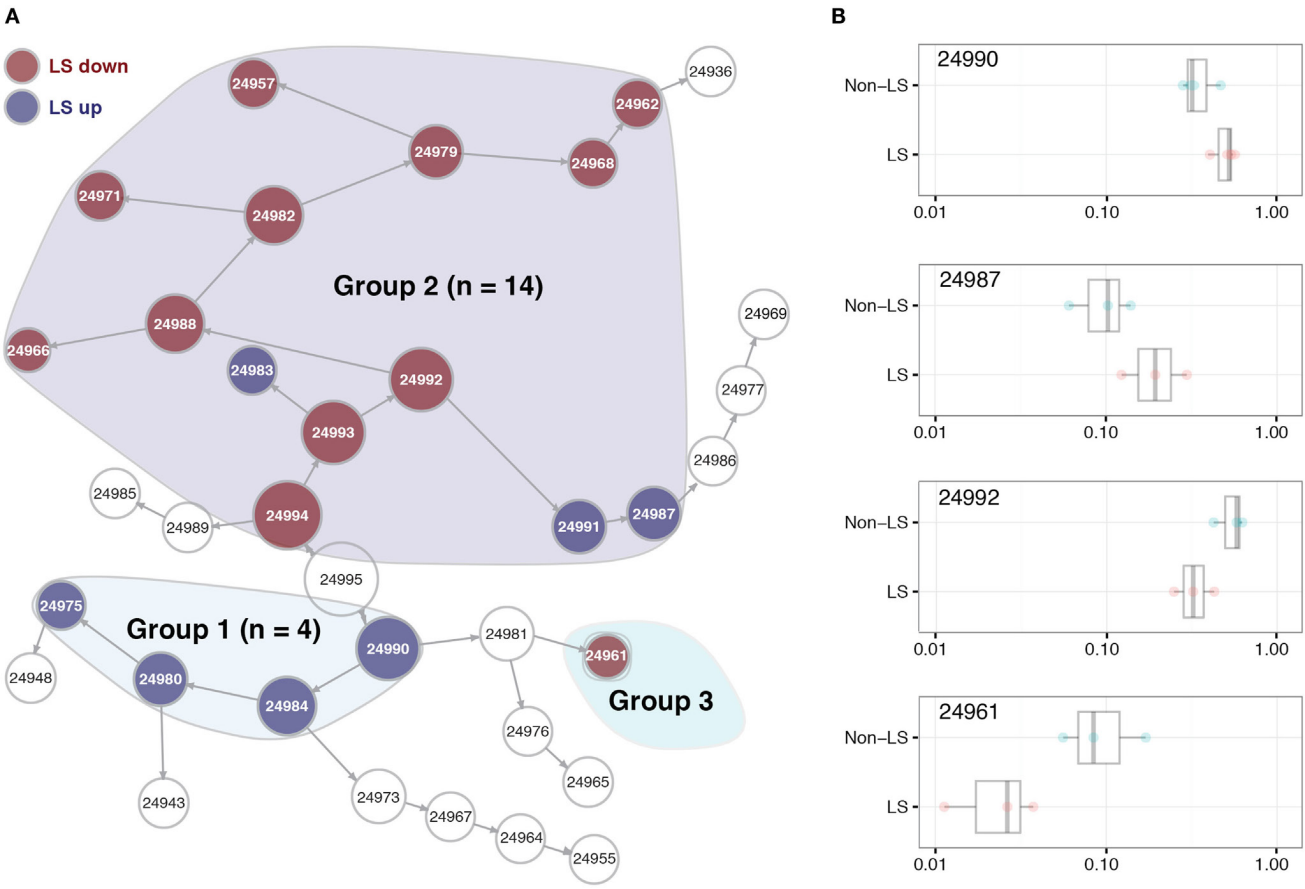
Significant differences in CD4^+^ T cell patterns between Löfgren’s syndrome (LS) and non-LS. **(A)** Citrus network tree visualizing the hierarchical relationship between identified bronchoalveolar lavage fluid CD4^+^ T cell populations in LS (*n* = 4) and non-LS (*n* = 4). Circle size reflects number of cells within a given cluster. Clusters differing significantly in abundance between the two conditions are divided into three main groups (highlighted). Populations more abundant in LS are indicated in blue, while those more abundant in non-LS are marked in red. **(B)** Citrus-generated box plots for four representative and differentially regulated populations in LS and non-LS, illustrative of all three cluster groups shown in panel **(A)**. Cluster abundances are shown on a logarithmic scale for clusters 24990, 24987, 24992, and 24961, respectively, annotated according to panel **(A)**. All differences in abundance are significant at FDR < 0.25.

Within the BALF CD4^+^ T cell population, we identified 19 clusters of cells whose abundance differed significantly between LS and non-LS patients. Seven of these were more abundant in LS, while 12 primarily appeared in non-LS (Figure 1A). Hierarchical clustering showed these 19 populations to assemble in three distinct groups, where clusters in group 1 were dominated by cells from LS patients and defined by a significant reduction in adhesion molecule CD44 compared to background cells (i.e., all CD4^+^ T cells). In group 2, clusters were distinguished by effector cell phenotypes, and in the case of LS-dominated populations, expression of TCR variable chains Vα2.3 and/or Vβ22. The sole cluster in group 3 showed an elevation in CD57 expression and was significantly reduced in LS patients (Figures 1A,B).

### Regulatory Elements are More Pronounced in LS CD4^+^ T Cells

**Figure 2:**
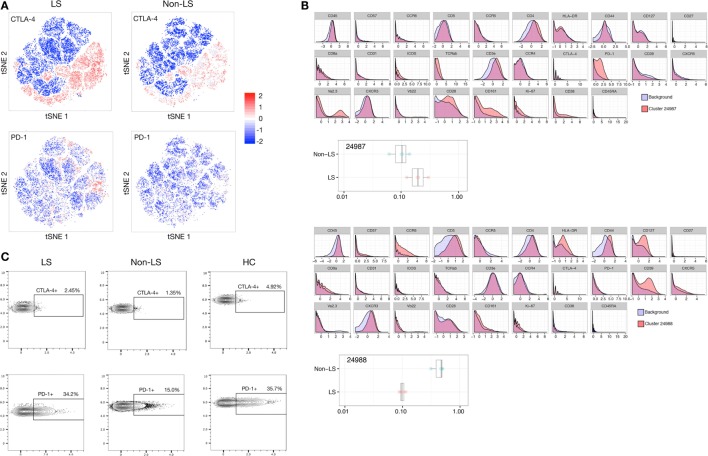
Regulatory elements dominate in Löfgren’s syndrome (LS) CD4^+^ T cells. **(A)** *t*-stochastic neighborhood embedding (*t*-SNE) plots visualizing distribution and intensity of CTLA-4 and PD-1 expression in LS and non-LS CD4^+^ T cells, respectively, following clustering of all samples combined and subsequent separation by condition. **(B)** Citrus-generated histograms showing significant changes in marker distributions in individual clusters (red) compared to background total CD4^+^ T cells (blue). Representative LS- and non-LS-specific populations are exemplified by clusters 24987 and 24988, respectively. **(C)** Representative FlowJo contour plots manually gated for single, live (DNA^+^ Cisplatin^−^), CD3^+^, CD4^+^, CD8^−^ T cells in LS and non-LS patients, respectively, showing expression of CTLA-4 and PD-1 in the two patient groups, as well as in a bronchoalveolar lavage fluid sample from a healthy control (HC).

Most notably, the regulatory profile of CD4^+^ T cells was markedly enhanced in LS patients. Citrus analysis and subsequent *t*-SNE visualization revealed the expression of immune-inhibitory receptors CTLA-4 and PD-1, as well as co-stimulator ICOS to be significantly elevated in LS CD4^+^ T cells compared to background CD4^+^ T cells. Most prominently, a twofold to threefold increase in PD-1 expression was observed in LS patients compared to background, while such a shift was virtually absent in non-LS patients (Figures 2A,B). Manual gating of cells using FlowJo revealed higher frequencies of CTLA-4^+^ (4.9%) and PD-1^+^ (35.7%) CD4^+^ T cells in BALF from a healthy individual compared to sarcoidosis patients of both phenotypes. Notably, however, CD4^+^ T cells from LS patients displayed higher median expression of both receptors (CTLA-4^+^ 1.8%, PD-1^+^ 27.8%) compared to non-LS cells (CTLA-4^+^ 1.4%, PD-1^+^ 14.6%), indicating an overall downregulation of these receptors in disease, but also that regulatory capacity is retained to a greater extent in LS lungs (Figure 2C; Table 3).

**Table 3:** Summary of CTLA-4 and PD-1 expression in bronchoalveolar lavage fluid (BALF) CD4^+^ T cells.

Diagnosis	CTLA-4^+^	PD-1^+^
Löfgren’s syndrome (LS) patient 1	1.55	19.10
LS patient 2	1.69	34.20
LS patient 3	2.45	51.50
LS patient 4	1.88	21.30
**Median**	1.79*	**27.75#**
Non-LS patient 1	1.43	15.00
Non-LS patient 2	1.54	13.50
Non-LS patient 3	1.29	14.20
Non-LS patient 4	1.35	33.00
**Median**	1.39*	**14.60#**
HC	4.90	35.70

Percentages of CTLA-4^+^ and PD-1^+^, respectively, CD4^+^ T cells in BALF from individual LS and non-LS patients, as well as one healthy control (HC), as determined by manual gating of single, live (DNA^+^Cisplatin^−^), CD3^+^, CD4^+^, CD8^−^ T cells in FlowJo X software. Comparison of median receptor expression in the respective patient groups by this method confirmed higher median expression of CTLA-4 (*p = 0.0286) and PD-1 (^#^*p* = 0.1143) in LS patients compared to non-LS (all comparisons by the non-parametric Mann–Whitney *U* test).

### Non-LS CD4^+^ T Cells Consistently Exhibit Effector Profiles

**Figure 3:**
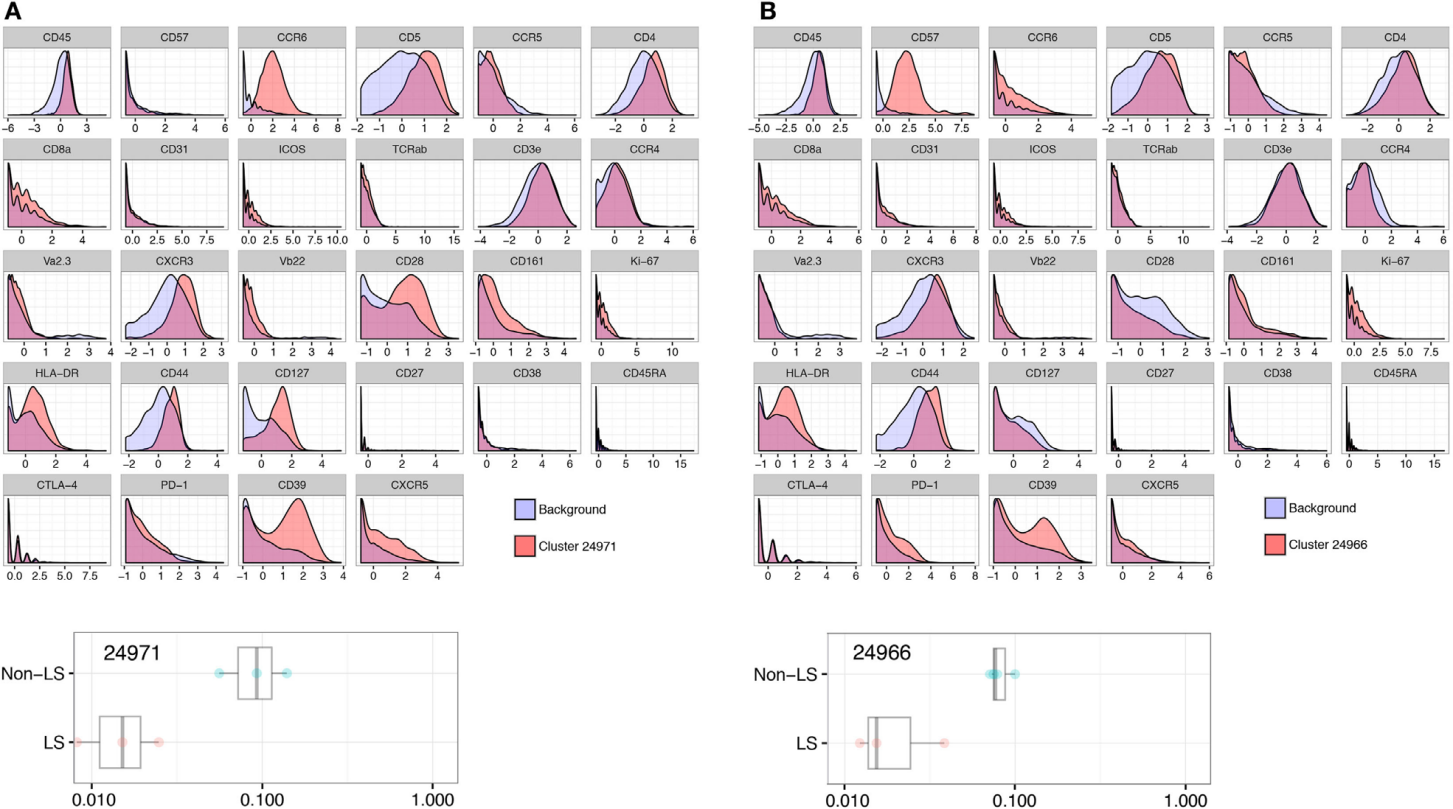
Non-Löfgren’s syndrome (non-LS) CD4^+^ T cells exhibit a pronounced effector profile. Enhanced influence of HLA-DR, CD127, and CD39 in non-LS CD4^+^ T cells as visualized by Citrus-generated histograms. A representative cluster (24971) abundant in non-LS patients is shown in panel **(A)**, exemplifying the significantly higher expression of these effector markers in non-LS. Similarly, cluster 24966 illustrates elevated expression of CD57 in one of two clusters prominent in non-LS **(B)**.

In clusters more abundant in non-LS patients, HLA-DR, CD127, and CD39 were all significantly elevated compared to background CD4^+^ T cells, consistently displaying a twofold, or in some cases nearly threefold, increase (Figure 3A). In addition, two clusters (24961 and 24966) showed a significant increase in CD57 expression that was not detected in LS-dominated clusters (Figure 3B).

### Adhesion Marker CD44 is Reduced in LS Compared to Non-LS CD4^+^ T Cells

**Figure 4:**
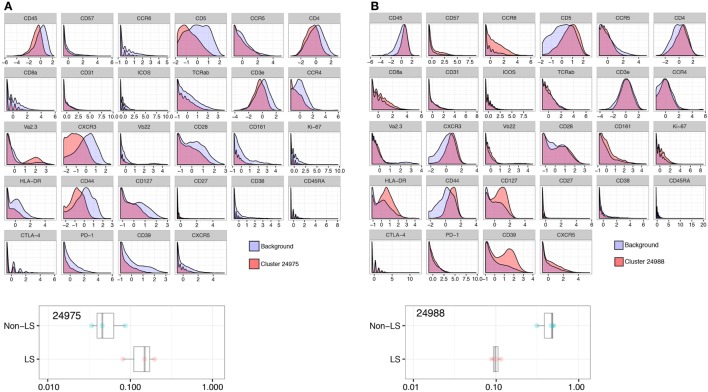
Adhesion marker CD44 is consistently reduced in Löfgren’s syndrome (LS). Expression of CD44 was significantly reduced in clusters more abundant in LS, as exemplified by cluster 24975 **(A)**, compared to non-LS **(B)** (cluster 24988).

Expression of the adhesion molecule CD44, believed to influence granuloma formation and persistence, was significantly reduced in LS CD4^+^ T cells with an expression onefold to twofold lower than background (Figure 4A). This reduction was consistent across all LS clusters but most prominent in group 1 cells (Figure 1A). Conversely, non-LS cells showed a shift in CD44 expression of similar magnitude, but opposite direction (Figure 4B). The differentially regulated marker expressions that characterize LS and non-LS are summarized in Table 4.

**Table 4:** Summary of differentially regulated marker expression.

Marker	Löfgren’s syndrome (LS)	Non-LS
CTLA-4	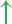	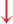
PD-1	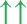	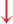
ICOS	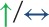	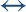
CD44	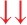	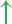
HLA-DR	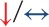	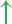
CD127	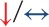	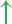
CD39	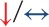	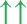
CD57	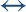	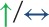

*Arrows indicate statistically significant changes in expression of markers distinctive of LS and non-LS, respectively, as determined by Citrus analysis. Arrow directionality is based on fold change compared to background CD4^+^ T cells, as follows: 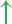, elevated expression (fold change ≤ 2), 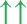, elevated expression (fold change ≥ 2), 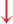, reduced expression (fold change ≤ 2), 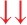, reduced expression (fold change ≥ 2), 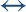, unaltered expression, /↔, expression elevated or reduced in certain clusters while unaltered in others*.

### TCR Vα2.3^+^CD4^+^ T Cells in LS are Functionally Heterogeneous

**Figure 5:**
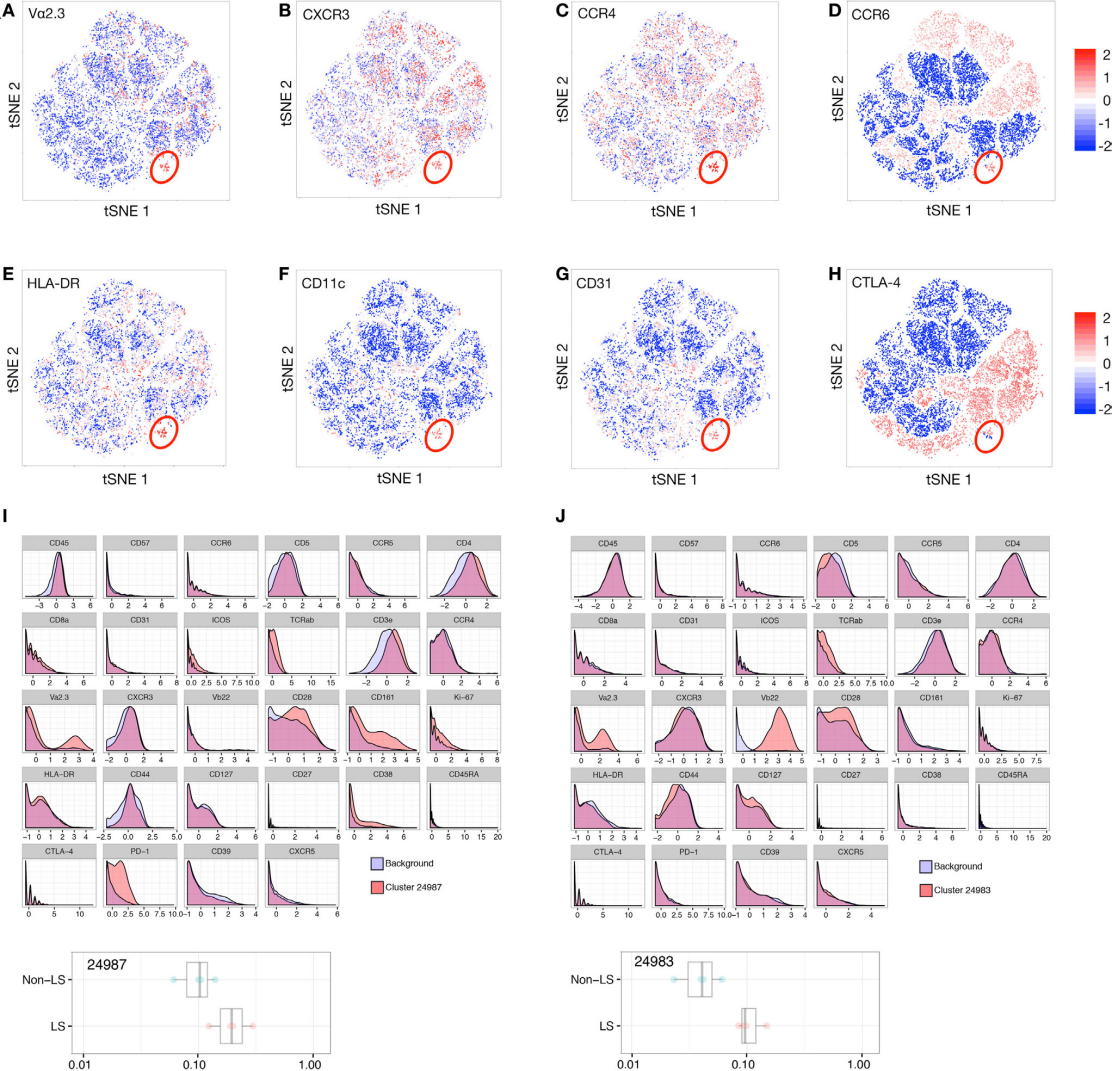
T cell receptor (TCR) Vα2.3^+^ cells in Löfgren’s syndrome (LS) constitute a functionally heterogeneous population. *t*-stochastic neighborhood embedding (*t*-SNE) plots filtered for expression of Vα2.3 **(A)**, CXCR3 **(B)**, CCR4 **(C)**, CCR6 **(D)**, HLA-DR **(E)**, CD11c **(F)**, CD31 **(G)**, and CTLA-4 **(H)** in LS bronchoalveolar lavage fluid cells. The main Vα2.3^+^ cluster is highlighted in red. Citrus comparison of Vα2.3^+^Vβ22^−^ **(I)** and Vα2.3^+^Vβ22^+^ **(J)** clusters (24987 and 24983, respectively) reveals distribution of effector (e.g., CD127, CD28) and regulatory markers (e.g., PD-1, ICOS) to vary with TCR expression.

By *t*-SNE visualization, a small cluster defined by high expression of TCR segment Vα2.3 was identified in LS patients (Figure 5A). Interestingly, this cluster concomitantly expressed chemokine receptors CXCR3, CCR4, and CCR6 (Figures 5B–D), as well as high levels of activation marker HLA-DR (Figure 5E), CD11c, associated with T cell differentiation and migratory potential (Figure 5F), and CD31, involved in leukocyte adhesion and a marker of recent thymic emigrant cells (Figure 5G). Expression of regulatory markers, e.g., CTLA-4 (Figure 5H), varied with TCR expression. Citrus comparison of Vα2.3^+^Vβ22^−^ (Figure 5I) and Vα2.3^+^Vβ22^+^ (Figure 5J) clusters (24987 and 24983, respectively) revealed abundance of both populations to be significantly increased in LS compared to non-LS patients, but the distribution of receptors associated with cell activation and effector functions (CD127, CD28, CD161) and immune regulation (PD-1, CTLA-4, ICOS) molecules, respectively, to differ between the two Vα2.3^+^ clusters. Judging by the network tree presented in Figure 1A, these two clusters also appear to have arisen from different origins.

### Overlapping Expression of CD8 and Ki-67 in Both LS and Non-LS

**Figure 6:**
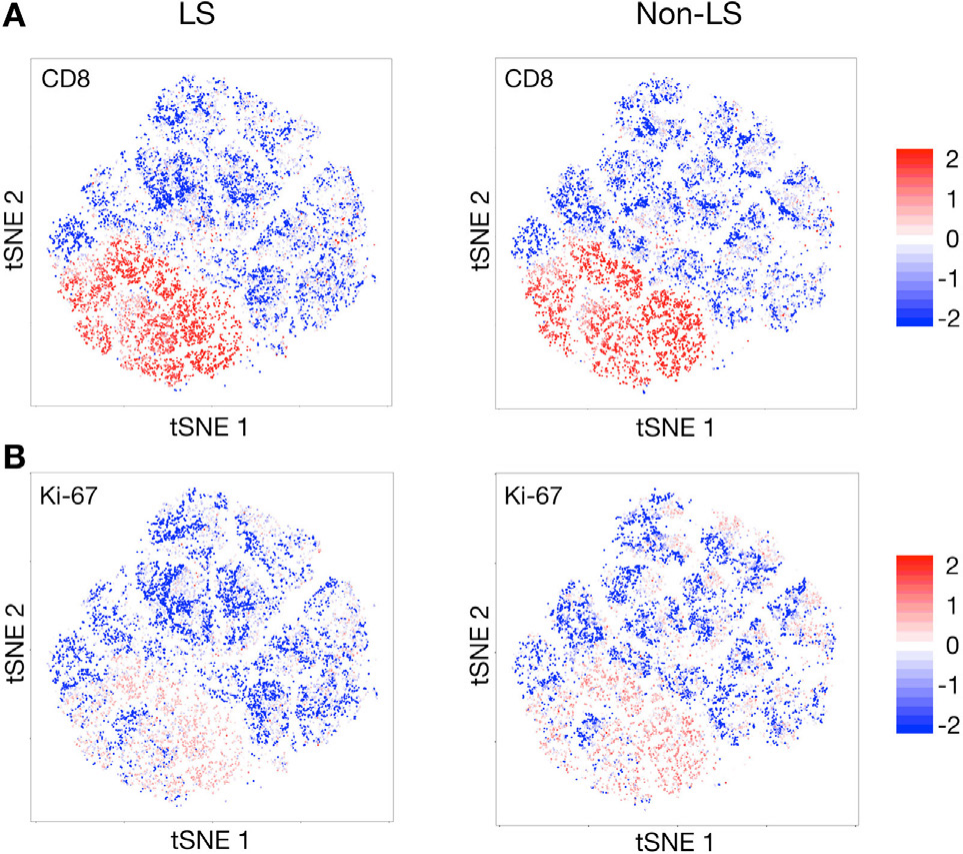
Near-complete overlap of CD8 and Ki-67 expression. *t*-stochastic neighborhood embedding (*t*-SNE) plots revealing an almost perfect overlap in expression of CD8 **(A)** and Ki-67 **(B)**, the latter being a marker of actively proliferating cells, in both Löfgren’s syndrome (LS) and non-LS patients.

Previous studies and the established diagnostic criterion of an elevated BALF CD4/CD8 ratio have hitherto assumed CD4^+^ T cells to be the driving force of disease in sarcoidosis. In both patient groups, however, expression of Ki-67, representing actively proliferating cells, and CD8 coincided remarkably well (Figures 6A,B), indicating an active role of CD8^+^ T cells in sarcoid inflammation. A significant increase in Ki-67 expression could also be observed for group 2 CD4^+^ T cell clusters (Figure 1A), i.e., those primarily exhibiting an effector profile, but much less pronounced than for CD8^+^ T cells.

## Discussion

While marked differences in clinical presentation and outcome have long defined LS and non-LS, comprehensive genetic disparities were only recently demonstrated (7), and the immunological mechanisms driving these two forms of sarcoidosis are still incompletely understood. To address key characteristics of CD4^+^ T cells in LS and non-LS in an unbiased fashion, we subjected BALF cells from two genetically and clinically distinct patient groups to mass cytometry and cluster analysis, identifying cell populations of significantly different abundance in the two disease phenotypes. On the individual marker level, significant changes were most notably observed for inhibitory receptors CTLA-4 and PD-1, adhesion marker CD44, effector and activation markers HLA-DR, CD127 and CD39, as well as a range of markers associated with cells expressing TCR variable segments Vα2.3 and/or Vβ22. These findings connect immunological profiles to established clinical characteristics of disease, and the identified novel markers may allow for both prognostic evaluation and more precise differentiation between sarcoidosis subtypes. Despite sample size being limited by methodological requirements and patient availability, these statistically robust differences further argue for LS and non-LS to be considered as immunologically distinct conditions.

Believed to influence tolerance induction and long-term tolerance maintenance, respectively, inhibitory receptors CTLA-4and PD-1 act to constrain development of autoimmunity. Notably, anti-CTLA-4 and anti-PD-1 treatments have the potential to initiate or exacerbate sarcoid-like inflammation (21, 22), reinforcing the notion of altered immune activation and possibly an autoimmune component in sarcoidosis. Broos et al. also previously found reduced CTLA-4 expression on sarcoidosis T cells compared to healthy individuals, suggestive of a shift in balance between immune activation and regulation that results in exacerbated T cell activity and, at length, chronic inflammation, and tissue damage (23). In this study, a similar reduction in CTLA-4 and PD-1 expression could be observed in sarcoidosis compared to a healthy individual. Among sarcoidosis patients, however, expression of both receptors was primarily found in LS CD4^+^ T cells, indicating a higher degree of immune self-restriction compared to non-LS patients. In addition, ICOS expression was significantly increased in some, though not all, LS clusters compared to background CD4^+^ T cells. Elevated ICOS expression has previously been demonstrated in LS (24) and could associate with the higher IL-10 production seen in these patients (25, 26). From a clinical perspective, LS patients in general and HLA-DRB1*03^+^ individuals in particular have a favorable prognosis, and in our opinion, it is reasonable to believe that superior regulatory capacity of CD4^+^ T cells in LS compared to non-LS influences the characteristic resolving disease course. Accordingly, it is interesting to speculate that stimulation of regulatory elements could have therapeutic potential in non-LS patients prior to progression to a chronic disease state.

In contrast, compared to background BALF CD4^+^ T cells from non-LS patients displayed an elevated expression of activation markers HLA-DR, CD127, and CD39 consistent across all clusters. Polymorphisms in the *CD127* (*IL-7R*) gene are known risk factors for sarcoidosis development (27), and the IL-7 receptor is crucial during T cell activation, homeostasis, and differentiation. Importantly, it is differentially expressed on effector and regulatory T cells (Tregs), with the latter being defined by low levels of this receptor. The lack of CD127 in LS-dominated clusters, combined with the expression of CTLA-4 and PD-1, suggests immune regulation to be maintained in LS CD4^+^ T cells, while the balance is shifted in favor of effector functions in non-LS CD4^+^ T cells. The role of CD39 in this context is less established, but CD39^+^CD4^+^ T cells have been proposed as inducers of T cell proliferation and cytokine production (28). Moreover, a recent study showed reduced suppressive function of CD39^+^ compared to CD39^−^ Tregs (29), implicating dysfunctional regulatory mechanisms particularly in the pathogenesis of non-LS. Notably, CD39^+^ cells have also been found in sarcoid granulomas (30).

While expression of CD57 is most commonly associated with NK cells, its presence has been documented in T cells with a more advanced differentiation state, reduced replicative capacity but retained cytokine production, particularly of IFNγ (31–33), and possibly cytotoxic functions (34). In the present study, two clusters, both of which more abundant in non-LS patients, demonstrated elevated CD57 expression compared to background CD4^+^ T cells. Higher IFNγ production in non-LS patients has been demonstrated previously (25, 26), and could possibly be at least partly attributed to such terminally differentiated cells with cytotoxic potential.

The adhesion molecule CD44 has been detected on lymphocytes in granulomas of both Crohn’s disease and pulmonary sarcoidosis, and suggested to affect their homing and activation, as well as macrophage differentiation (35). Interestingly, in the present study, CD44 was consistently reduced in LS CD4^+^ T cell clusters compared to background, while the opposite was observed in non-LS clusters. As T cells are essential for maintenance of granulomatous structures, reduced adhesive capacity of T cells may well play a role in spontaneous resolution of granulomas, thereby influencing disease resolution in LS. Of note, any clinically relevant differences between LS and non-LS granulomas have yet to be investigated, but the prospect that alternate granulomatous development might ultimately affect disease outcome is worth considering. Recently, involvement of the mTORC1 pathway was proposed to promote disease progression in sarcoidosis (36). Intriguingly, mTORC1 complexes can be inhibited by CD44 blockade (37), suggesting a dual role for CD44 in granuloma persistence, acting both during cell–cell adhesion and signal transduction.

The recently noted clonality of Vα2.3^+^Vβ22^+^CD4^+^ T cells (8) indicates them to be a driving force in HLA-DRB1*03^+^-mediated antigen recognition. The current study further suggests that cells expressing Vα2.3 can initiate and/or act within different immune pathways. Moreover, expression of Vα2.3 together with Vβ22 appear to associate with effector-like markers, while Vα2.3^+^ populations with other Vβ segment pairings preferentially expressed markers associated with immune regulation, such as PD-1 and ICOS. As hierarchical clustering suggests these clusters to arise from separate origins, it is plausible that several oligoclonal Vα2.3^+^ populations expand in the lungs in response to different epitopes derived from the same antigen, and that these cells serve discrete purposes during antigen recognition and clearance. Previous observations of both an effector phenotype of Vα2.3^+^ cells (5, 6, 8, 38), as well as reduced IFNγ and Th1 responses in LS patients (25, 26), could possibly be at least partly attributed to divergent TCRβ chain preferences. Expression of multiple chemokine receptors (CXCR3, CCR4, and CCR6) suggests that Vα2.3^+^CD4^+^ T cells might follow different migratory patterns compared to Vα2.3^−^CD4^+^ T cells, possibly allowing for more efficient interaction with other immune cells. The concurrent high expression of HLA-DR and CD31 might also signify their recent activation and ongoing homing to the site of antigen presentation. Activated, antigen-specific T cells expressing CD11c have been found to associate with both effector and regulatory profiles (39–41), and in this study, we show that CD11c^+^Vα2.3^+^ cells can express either co-stimulatory or co-inhibitory molecules. Alternatively, this functional discrepancy may reflect a transitional state of activated T cells following antigen recognition, implying that multiple stages of differentiation occur throughout the disease process. A gradual shift toward a more regulatory profile may thus be an inherent characteristic of LS lung CD4^+^ T cells, ultimately contributing to a better prognosis.

In both patient groups, the near-complete overlap of Ki-67 and CD8 expression conceivably points to a more pronounced activation of CD8^+^ T cells in sarcoidosis than previously appreciated. It is intriguing to speculate that lack of regulatory capacity in non-LS CD4^+^ T cells may result in unrestrained proliferation and increased cytotoxic activity of CD8^+^ T cells, which could promote tissue damage and chronic inflammation. In line with this hypothesis, a stronger cytotoxic response in non-LS CD8^+^ T cells was recently observed by Brighenti et al. (in preparation). Accordingly, tighter regulation of the CD8^+^ response by CTLA-4- and PD-1-expressing CD4^+^ T cells in LS patients could contribute to spontaneous resolution and absence of permanent tissue scarring.

Despite the differences observed, the low sample number is an obvious limitation to the study, as is the inability to perform validation experiments in the same cohort. Mass cytometry is a powerful method for detecting robust differences in small sample sizes, thus making it suitable for unbiased discovery studies. However, the cell number requirement is higher than for flow cytometry, which is a restraint when using clinical samples, and due to sample vaporization, no additional sorting or functional characterization experiments can be performed on the same cells. Moreover, established techniques such as flow cytometry or western blot have lower sensitivity and are, therefore, not ideal means of validation of mass cytometry data. Nevertheless, the current study demonstrates a novel approach to the study of CD4^+^ T cells in the lung and lays a foundation for future studies, ideally comprising larger groups of patients. The deliberately high FDR threshold for Citrus analysis enables identification of subpopulations that can be further explored in more stringent and comprehensive studies. Of note, several differentially regulated clusters in this study are derived from the same “parent” cluster and, therefore, exhibit similarities in marker expression, thus strengthening the accuracy of identified differences at the individual marker level despite a higher FDR. Future analyses should also aim to expand the current T cell panel to include markers of interest when investigating tissue-resident T cells, such as CD69, or for detailed delineation of Tregs, e.g., CD73 and CD25. Targeting of additional cytokine receptors and a broad range of transcription factors could also contribute to a more comprehensive subclassification of T cells and their role in different disease phenotypes.

## Conclusion

Together, these mass cytometry-based findings contribute new information on lung T cell differentiation and activation in two forms of sarcoidosis that may in time be considered as separate diseases. In LS, a stronger regulatory profile along with decreased adhesive capacity of CD4^+^ T cells, offer a stark contrast to the non-LS effector profile and a direct link to characteristic clinical symptoms. The demonstrated ability to detect significant differences at the molecular level, despite low sample numbers and limited availability of cells from the lungs, could be employed to further distinguish between clinical subtypes, e.g., based on presence of extra-pulmonary symptoms.

In conclusion, mass cytometry provides a powerful and sensitive means of understanding cellular mechanisms of disease that could prove especially valuable in heterogeneous conditions such as non-LS sarcoidosis, and markers that differentiate disease phenotypes could potentially be exploited for future therapeutic purposes in non-resolving disease.

## Ethics Statement

This study was carried out in accordance with the recommendations of Karolinska Institutet, Karolinska University Hospital and the Stockholm County Regional Ethical Committee with written informed consent from all subjects in accordance with the Declaration of Helsinki. The protocol was approved by the Stockholm County Regional Ethical Committee.

## Data Reposition

Mass cytometry data in the form of FCS files can be accessed from FlowRepository (https://flowrepository.org/) *via* accession number FR-FCM-ZY9W.

## Author Contributions

Conception and study design: YK, AA, and JG. Sample collection: YK and AE. Mass cytometry panel development, optimization, and implementation: TL, JM, and PB. Data pre-processing and cluster analysis (Citrus, ACCENSE): YC and PB. Data visualization (*t*-SNE): YK. Interpretation and assessment of clinical parameters: YK, AA, and JG. Manuscript preparation: YK, AA, and JG. Critical reading and intellectual assessment of manuscript: YK, TL, AE, PB, AA, and JG. All authors read and approved the final manuscript.

## Conflict of Interest Statement

The authors declare that the research was conducted in the absence of any commercial or financial relationships that could be construed as a potential conflict of interest.
